# Atmospheric Pollution Monitoring in Urban Area by Employing a 450-nm Lidar System

**DOI:** 10.3390/s18061880

**Published:** 2018-06-08

**Authors:** Zheng Kong, Zhi Liu, Lishan Zhang, Peng Guan, Limei Li, Liang Mei

**Affiliations:** School of Optoelectronic Engineering and Instrumentation Science, Dalian University of Technology, Dalian 116024, China; KongZheng@mail.dlut.edu.cn (Z.K.); liuzhi@mail.dlut.edu.cn (Z.L.); zlss@mail.dlut.edu.cn (L.Z.); 836346519@mail.dlut.edu.cn (P.G.); llm0424@mail.dlut.edu.cn (L.L.)

**Keywords:** lidar, remote sensing and sensors, air pollution monitoring, aerosol detection, diode lasers

## Abstract

In past decades, lidar techniques have become main tools for atmospheric remote sensing. However, traditional pulsed lidar systems are relatively expensive and require considerable maintenance. These shortcomings may be overcome by the development of a blue band Scheimpflug lidar system in Dalian, Northern China. Atmospheric remote measurements were carried out for 10 days in an urban area to validate the feasibility and performance of a 450-nm Scheimpflug lidar system. A 24-h continuous measurement was achieved in winter on a near horizontal path with an elevation angle of about 6.4°. The aerosol extinction coefficient retrieved by the Fernald-inversion algorithm shows good agreement with the variation of PM10/PM2.5 concentrations recorded by a national pollution monitoring station. The experimental result reveals that the linear ratio between the aerosol extinction coefficient and the PM10 concentration under high relative humidity (75–90%) is about two-times that in low relative humidity (≤75%) when the PM10 concentrations are less than 100 µg/m^3^.

## 1. Introduction

Air pollution, especially anthropogenic particulate pollution has been a serious environmental problem in China since the rapid development of industrialization and urbanization during past decades [1,2]. Morbidity and mortality of cardiovascular and respiratory diseases significantly increase during recent years due to severe hazes in China [3]. Moreover, the absorbing and scattering effects of particulate matters can also influence earth’s radiation budget and global climates [4,5,6,7]. Atmospheric particles and gas pollutants monitoring appears particularly important and urgent. The Chinese Environmental Council recently released an air pollution prevention and control action plan, aiming at reducing inspirable particle concentrations by 20% from 2012 to 2017.

Remote sensing of atmospheric aerosols is traditionally achieved by detecting backscattered light from nanosecond laser pulses emitted into the atmosphere. Atmospheric backscattering or extinction coefficient, which are correlated to particle concentrations, can be retrieved from the backscattering lidar signal according to the Fernald-Klett inversion method, etc. [8,9,10,11,12]. As a remote sensing technique, lidar is capable of real-time monitoring of atmospheric particle distribution and variation in large areas, e.g., urban area pollution monitoring, while point monitoring instruments are only able to measure local concentrations. Extensive work has been pursued by utilizing atmospheric lidar techniques for atmospheric pollution monitoring [13,14,15,16,17,18,19]. Scott M. Spuler et al. presented a field-deployable lidar system based on an eye-safe laser (1.54 µm) with a blind range of about 500 m [14]. T. Y. He et al. demonstrated a lidar system employing a 532-nm pulsed Nd:YAG laser, which was capable of tracking two-dimensional particle distribution with an angular resolution of 0.1° [15]. However, the incomplete overlap region is rather large, it extends as far as 0.8 km. Recently, C.-W. Chiang, et al., developed a mobile and portable scanning lidar system for profiling pollutants in lower troposphere [20]. The lidar systems discussed above mainly employ high-cost and sophisticated laser sources, e.g., Nd:YAG lasers. Besides, a data acquisition unit with high sampling rate and large dynamic range is often used to achieve high spatial resolution as well as long detection range. The relatively robust and inexpensive ceilometers, e.g., Jenoptik CHM15k, are now broadly deployed for cloud and aerosol layer detections [21,22]. However, the signal-to-noise ratio (SNR) of the atmospheric lidar signal decreases to about 1 at 4 km during daytime with 30-min signal averaging due to low pulse energy [22]. Furthermore, a common issue of the pulsed lidar systems is the large blind range due to incomplete geometric overlap between the transmitter and the receiver, which could be partially solved by measuring the overlap function or adjusting the alignment for near and far range.

Recently, the Scheimpflug lidar (SLidar) technique, based on the Scheimpflug principle, has been demonstrated successfully for atmospheric remote sensing [23,24,25]. The backscattering light of the entire aerosol volume illuminated by the transmitted laser beam can be clearly focused on a tilted image sensor, if the image sensor plane, the lens plane and the laser beam (object) plane intersect into a single line—satisfying the Scheimpflug principle. The pixels of the Complementary Metal Oxide Semiconductor (CMOS)/ Charge-coupled Device (CCD) sensors correspond to the distances of the illuminated volume. The SLidar technique significantly reduces system complexity and cost by utilizing high-power continuous-wave laser diodes and highly integrated CMOS/CCD sensors. Besides, the SLidar technique can achieve a short blind range less than 100 m or even up to 30 m by employing a large-area rectangular senor. Small-scale 450-nm Scheimpflug lidar system has been recently implemented, but mainly for applications in short range, such as aquatic ecosystem studies [26] and oil pollution monitoring [27]. An all-time (24-h) operating blue-band SLidar system has not been implemented for atmospheric pollution monitoring, in spite of the great interest for atmospheric aerosol monitoring and differential absorption measurement of NO_2_ distribution [28,29,30,31].

This work aims at developing a SLidar system operating in the blue region by employing a high-power 3.5-W multimode 450-nm laser diode as the laser source. Twenty-four-hour continuous atmospheric measurements were performed for 10 days in Dalian, Northern China during haze and clean weather conditions on a near horizontal path. The Fernald-inversion algorithm is also applied for the time-range map retrieval of the aerosol extinction coefficient. The performance and feasibility of employing the 450-nm SLidar system for atmospheric pollution monitoring is also validated by comparing the experimental results with the PM2.5/PM10 concentrations measured by a national pollution monitoring station under various atmospheric conditions.

## 2. Experimental Setup

### 2.1. The 450-nm Scheimpflug Lidar (SLidar) System

**Figure 1:**
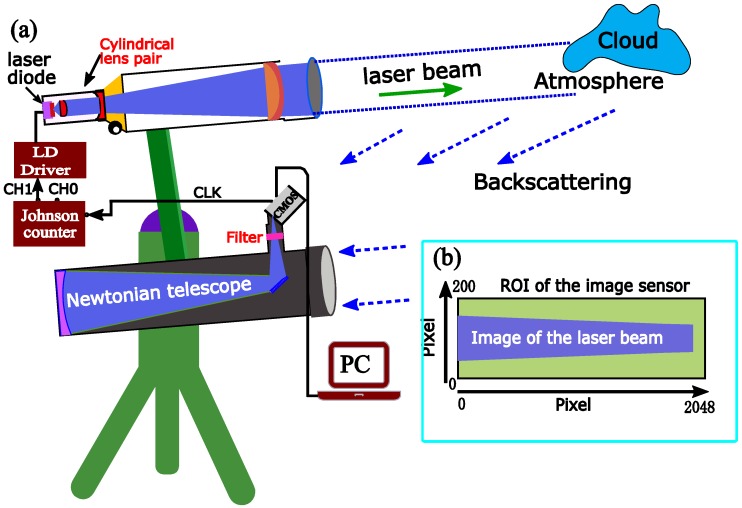
(**a**) Optical layout of the Scheimpflug lidar system, the figure is not to scale. The slow axis is placed in the Scheimpflug plane (paper plane), while the fast axis is placed perpendicular to the paper plane. The laser beam along the fast axis is collimated by both the cylindrical lens pair and the F6 lens, which are confocal. (**b**) Recorded image of the laser beam by the Complementary Metal Oxide Semiconductor (CMOS) image sensor (on image).

The optical layout and the primary specifications of the SLidar system are shown in Figure 1 and Table 1, respectively. A high-power multimode continuous-wave 450-nm laser diode and a CMOS image sensor are employed as the laser source and the detector, respectively. The high-power laser diode with a TO9 package is housed by a customized mount for fine case temperature controlling. Laser diodes commonly have large divergence and an elliptical beam shape. For instance, the divergences of the 450-nm laser diode are about 46° along the fast axis (1/e^2^) and 14° along the slow axis (1/e^2^). The emission facet is about 1 µm (fast axis) × ≈ 50 µm (slow axis). The large divergence leads to a very low geometrical transmission efficiency particularly in the fast axis when employing large f-number optics. In this work, a cylindrical lens pair is employed to reduce the divergence of the laser beam in fast axis before collimated by a long focal refractor telescope (F6, f = 600 mm, ∅ = 100 mm). The laser beam along the fast axis is first collimated by a convex cylindrical lens with an acceptance angle of ±19° and then a concave cylindrical lens, which are confocal. As a result, the emission facet along the fast axis is magnified to form an enlarged virtual image, while the divergence is reduced to about ±4.6° that matches the acceptance angle of the F6 lens of the refractor telescope. The laser beam that has been enlarged in the fast axis is then collimated by the F6 lens and transmitted into atmosphere, while the slow axis of the laser diode is placed in the Scheimpflug plane (the plane of the optical layout). The geometrical transmission efficiency along the fast axis can be improved by a factor of 3 compared to the situation with only the F6 lens as the collimator.

Atmospheric backscattering light is collected by a 200-mm F4 Newtonian telescope. Although the linewidth of the 450-nm laser diode is about 2 nm, interference filters with similar bandwidth are not readily available. In this work, a 450-nm interference filter with a 10-nm full-width at half maximum (FWHM) is utilized to suppress the sunlight background radiation. The backscattering light from the probe volume is clearly focused on a 45° tilted CMOS sensor by the Newtonian telescope. The refractor telescope and the Newtonian telescope are mounted on an aluminum-alloy bar with approximately 806 mm separation to satisfy the Scheimpflug principle. The lidar system is mounted on an equatorial mount, allowing the adjustment of the observation angle. The laser diode is on/off modulated through the driving current. As shown in Figure 1a, the exposure signal of the CMOS camera is fed to a Johnson counter, where the on/off modulation signal is generated. Atmospheric background image (off image) as well as the backscattering image of the laser beam (on image) are thus captured alternatively in the region of interest (ROI: 2048 × 200 pixels) of the CMOS sensor. The on/off image pairs are vertically binned, respectively. The atmospheric background signal is then subtracted by signal interpolation to obtain a single lidar recording [25]. The raw lidar signal is obtained from the median average of a number of lidar recordings, e.g., 1000 times.

The exposure time of the CMOS camera is automatically changed during 24-h continuous measurements to optimize the SNR [32], e.g., 20 ms under full sunshine and 500 ms during nighttime. The averaging number for a single lidar curve is also changed in order to keep identical total measurement time for each lidar curve, e.g., 1000 times @20 ms exposure time and 40 times @500 ms exposure time. Thus, the total measurement time for a single lidar curve is approximately 45 s, including the time of measuring both the on/off images, as well as the data acquisition and transfer time. After signal averaging, the lidar signal is further de-noised by the Savitzky-Golay (S-G) filter with a frame length of 79 and an eight-order polynomial to eliminate the sunlight background noise and the noise of the CMOS sensor. Besides, signal resampling is performed for the lidar curve in the near range (85–700 m) by taking the weighted average of each 3-m subset signals [32].

### 2.2. Divergences of the Laser Beam

**Figure 2:**
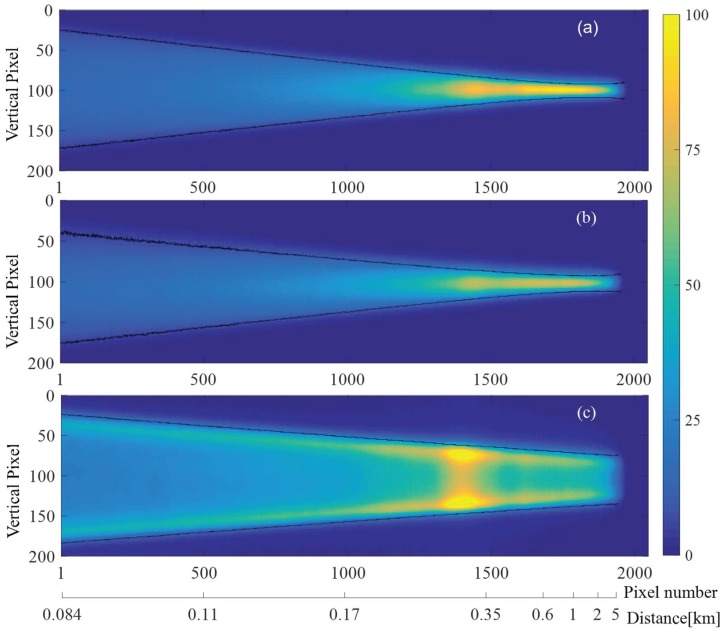
Images of the laser beam in atmosphere: (**a**) fast axis; (**b**) slow; and (**c**) fast axis are placed perpendicular to the Scheimpflug plane, respectively. The laser beam is collimated with the cylindrical lens pair and the F6 lens in figure (**c**). The black-solid curves indicate the half intensities of an image in vertical direction.

**Figure 3:**
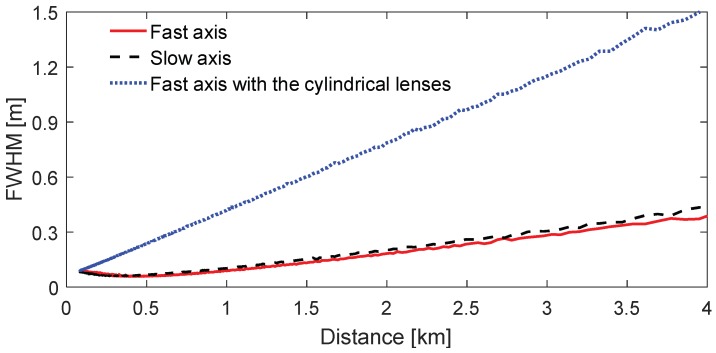
Full-width at half maximum (FWHM) of the laser beam in atmosphere along different axes. The blue-dot curve is obtained when the laser beam is collimated by the cylindrical lens pair and the F6 lens.

The divergences as well as the beam sizes of the transmitted laser beam should be minimized to achieve the best effective range resolution for the Scheimpflug lidar technique [25]. Atmospheric backscattering images were thus measured with 500-ms exposure time during nighttime to characterize the profile of the transmitted laser beam, as shown in Figure 2. The pixel-distance relationship can be calibrated by measuring the pixel position of the backscattering echo from a hard target (approximately 1 km away). When aligning the slow axis in the Scheimpflug plane (the plane of the optical layout), the CMOS sensor records the image of the transmitted laser beam along the fast axis (fast-axis image); vice versa. Figure 2a, b shows the laser images measured with only the F6 lens as the collimator. As can be seen, the fast-axis and slow-axis images are nearly the same, although the beam divergence and the chip size of the 450-nm laser diode along the slow and fast axes are quite different. The FWHM of the laser beam in atmosphere can be estimated from the width of the images according to the geometrical optics. The image width in different distance (pixel) can be obtained by finding the half maximum along vertical pixels, as illustrated by the black-solid lines in Figure 2. The width of the laser beam in atmosphere is estimated according to the lens equation. As shown in Figure 3, the beam width linearly increases with the measurement distance. The beam divergences along the fast and slow axes are identical, i.e., 0.1 mrad. Figure 2c shows the fast axis image when the transmitted laser beam is collimated by the cylindrical lens pair and the F6 lens. As can be seen, the image width in the later part of Figure 2c, corresponding to the far range, is much larger compared to the situation when the laser beam is collimated with only the F6 lens. The divergence along the fast axis is increased to about 0.36 mrad.

## 3. Measurements

**Figure 4:**
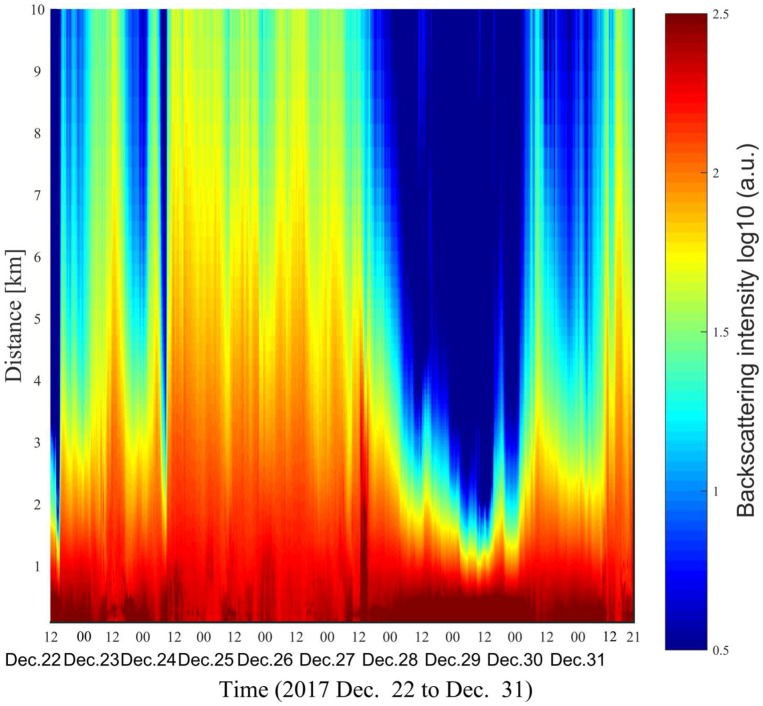
Time-space map of the atmospheric backscattering signal measured from 22 December to 31 December 2017.

Atmospheric measurements were performed in Dalian city on a near horizontal path from 22 December to 31 December 2017. A severe haze occurred on 28 to 29 December. Atmospheric parameters such as relative humidity, temperature, wind speed, and PM2.5/PM10 concentration were reported once an hour by a local national pollution monitoring station, located at 2.5 km away from the lidar system in the southwest-south direction. The elevation angle of the lidar system is about 6.4°, limited by the field of view of our laboratory. The pixel-distance relationship is calibrated by measuring the backscattering echo from a tall building located at about 971 m. As shown in Figure 2, the laser beam image can be fully captured, leading to a geometrical compressions factor of 1 even in the near range. Thus, the minimum measurement distance, which is then limited by the length of the CMOS sensor, can reach to about 85 m. Range correction is not required as the backscattering intensity of the SLidar technique does not decrease with the square of the measurement distance. The time-space map of the atmospheric backscattering signal is shown in Figure 4. The signal-to-noise ratios (SNRs) of the lidar curves are beyond 150 during daytime and 300–400 during night time in the measurement range of 85–150 m. The SNR generally increases with the decreasing of the sunlight background. However, it is finally limited by the photon-response non-uniformity (PRNU) noise of the CMOS sensor. Nevertheless, the SNR does not decrease with the square of the measurement distance. Although the near-range SNR is lower than that in conventional pulsed lidar techniques, the maximum measurement distance with SNR larger than 10 can still reach up to 7 km during daytime in clean atmospheric conditions. The maximum measurement range may be limited to 2–3 km during severe haze weather (PM10 ≈ 200 µg/m^3^), as can be seen from backscattering signals in the period from 28 to 29 December.

The aerosol extinction coefficient can be retrieved by the Fernald-inversion method, given by:

(1) $$\alpha_{a}(z)=-\frac{S_{a}}{S_{m}}\alpha_{\mathrm{mol}}(z)+\frac{P(z)\exp\left[2(\frac{S_{a}}{S_{m}}-1)\int_{z}^{z_{c}}\alpha_{m}(\zeta )d\zeta \right]}{\frac{P(z_{c})}{\alpha_{a}(z_{c})+\frac{S_{a}}{S_{m}}\alpha_{m}(z_{c})}+2\int_{z}^{z_{c}}P(\zeta )\exp\left[2(\frac{S_{a}}{S_{m}}-1)\int_{\zeta }^{z_{c}}\alpha_{m}(z^{\prime })dz^{\prime }\right]d\zeta }.$$

Here P(z) is the backscattering intensity at distance z, $S_{m}$, and $S_{a}$ are the molecular and aerosol lidar ratio, respectively, $\alpha_{m}(z)$ and $\alpha_{a}(z)$ are the molecular and aerosol extinction coefficients, respectively, $z_{c}$ is the calibration distance of the aerosol extinction coefficient and $\alpha_{a}(z_{c})$ is often referred to as the boundary value. The molecular lidar ratio and the extinction coefficient can be estimated from the atmospheric model, which can be considered as range-independent as the measurements were performed on a near horizontal path. The aerosol lidar ratio at 450 nm can be set to 50. The boundary value of $\alpha_{a}(z_{c})$ must be determined in order to retrieve the range-dependent aerosol extinction coefficient, as shown by Equation (1). The boundary value is retrieved by linearly fitting the log-scale lidar signal in a homogeneous subinterval range according to the Colis’ slope method. The retrieval distance of the boundary value is often in the far end to achieve a more stable solution for Equation (1). In this work, the maximum retrieval distance of the boundary value is set to 7 km. As the retrieval range is also limited by the SNR of the lidar signal, the boundary value is evaluated in the subinterval signal regions where the signal intensity is not less than 10 (SNR > 10).

## 4. Results and Discussions

**Figure 5:**
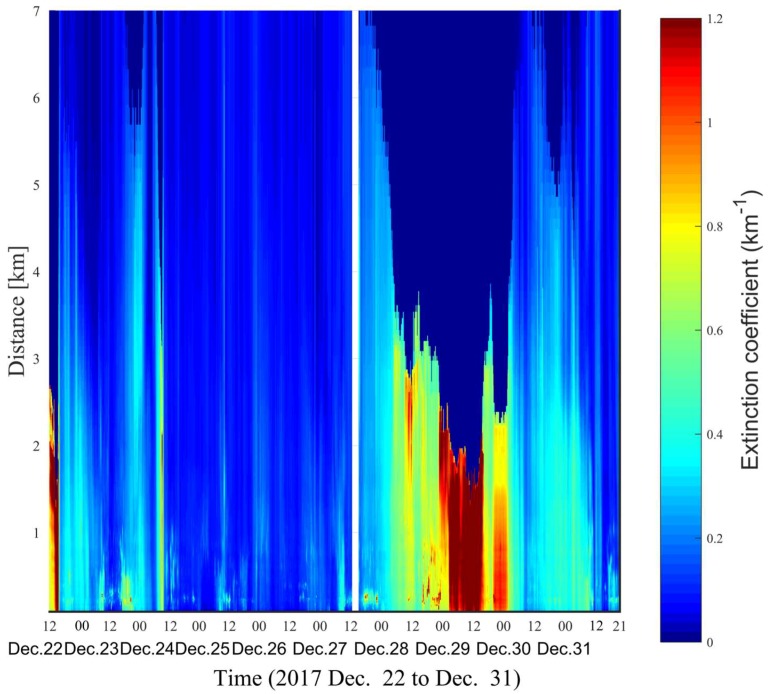
Aerosol extinction coefficient retrieved by the Fernald-inversion algorithm. The white-stripe area is the period with suspicious cloud appearing in the laser beam path.

The time-space map of the aerosol extinction coefficient is shown in Figure 5, from which the transportation and time-variation of atmospheric pollution can be readily observed. On 22 December, a severe haze was accumulating, while the north wind (5–8 m/s) blew away particulate matters at noon. The peak concentrations of PM2.5 and PM10 are 190 µg/m^3^ and 118 µg/m^3^, respectively, corresponding to an aerosol extinction coefficient of 1.35 km^−1^. In the early morning on 24 December, the particulate concentrations as well as the aerosol extinction coefficient suddenly increased in companion with the strong wind from the North (5–10 m/s). However, the pollution shortly disappeared. This implied that the suddenly appeared pollution was most likely due to the transportation of external pollution sources. After this period, the atmosphere is rather clean and homogeneous, and the PM2.5 and PM10 concentration were below 12 µg/m^3^ and 41 µg/m^3^ until the morning on 27 December. Nevertheless, local emissions due to traffic, road construction, etc., can still be observed in the near range between 85–1000 m, corresponding to an altitude below 100 m for an elevation angle of 6.4°. During this period, the aerosol extinction coefficient is also very low, about 0.1 km^−1^. Since the afternoon on 27 December, atmospheric pollution started to accumulate. In the meanwhile, the relative humidity also gradually increased, and reach up to 80% at midnight. The high relative humidity promoted the rapid growth of particulates, leading to very high concentrations of PM2.5 and PM10, i.e., 125 µg/m^3^ and 200 µg/m^3^, respectively. The aerosol extinction coefficient is about 1.7 km^−1^. Since the early morning on 30 December, the atmospheric pollution started to dissipate.

The aerosol extinction coefficient is highly relevant with the particulate concentration as has been discussed above. In order to compare the result measured by the lidar technique and the point monitoring station, the aerosol extinction coefficient should be averaged in both space and time. The spatial averaged value of the aerosol extinction coefficient can obtained from the ratio between the integral of the aerosol extinction coefficient along the measurement path and the total measurement range for each lidar signal:

(2) $$\alpha_{\mathrm{aer}}*=\frac{1}{z_{\max}-z_{\min}}\int_{z_{\min}}^{z_{\max}}\alpha_{\mathrm{aer}}(z)dz.$$

**Figure 6:**
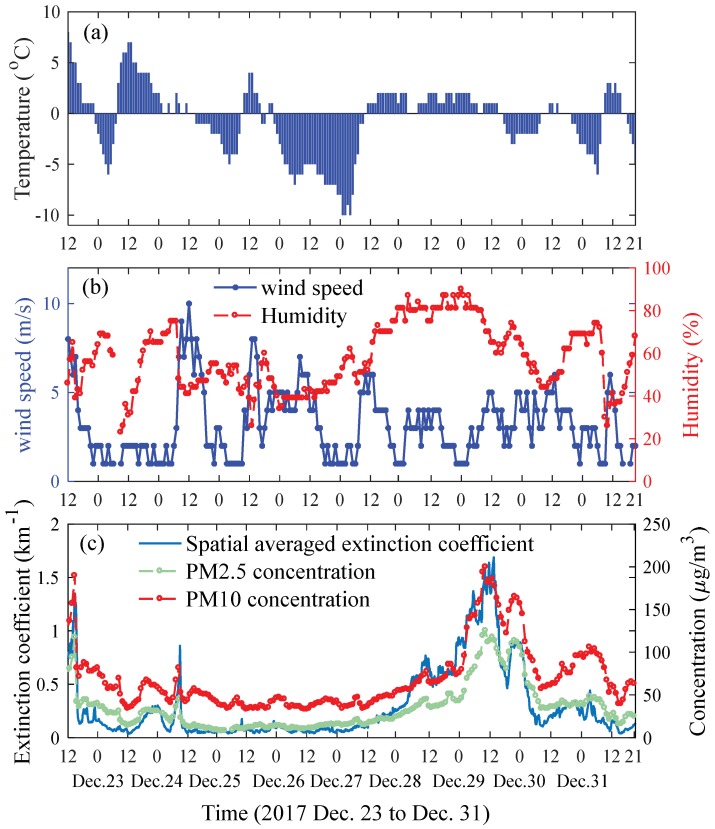
(**a**) Temperature; (**b**) wind speed and relative humidity; (**c**) atmospheric aerosol extinction coefficient retrieved from the lidar measurement and the particle concentrations reported by a local national pollution monitoring station. It should be noted that the spatial-averaged aerosol extinction coefficient is 10-times averaged in figure (**c**).

Here $z_{\max}$ and $z_{\min}$ are the maximum and minimum retrieval distances of the extinction coefficient for each lidar curve. The value of $z_{\max}$ can vary significantly under different weather conditions, while the value of $z_{\min}$ is nearly unchanged. The time-variation of the spatial averaged extinction coefficient together with the PM2.5/PM10 concentration is shown in Figure 6c. As can be seen, the variations of the extinction coefficient and the particulate concentrations are in good agreement.

**Figure 7:**
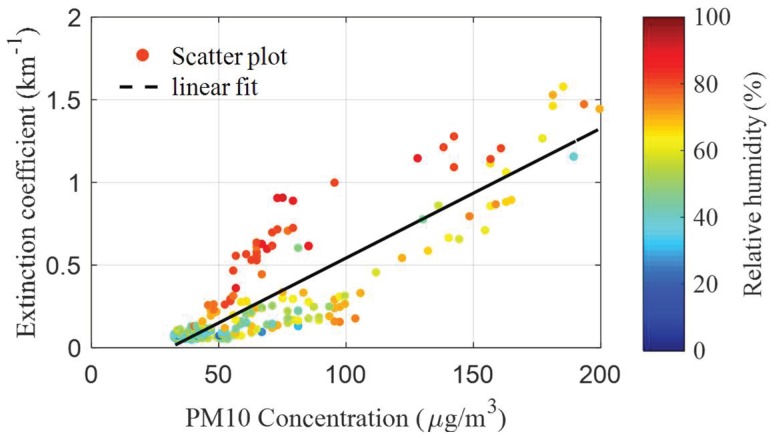
The relationship between aerosol extinction coefficient and the PM10 concentration under different relative humidities.

**Figure 8:**
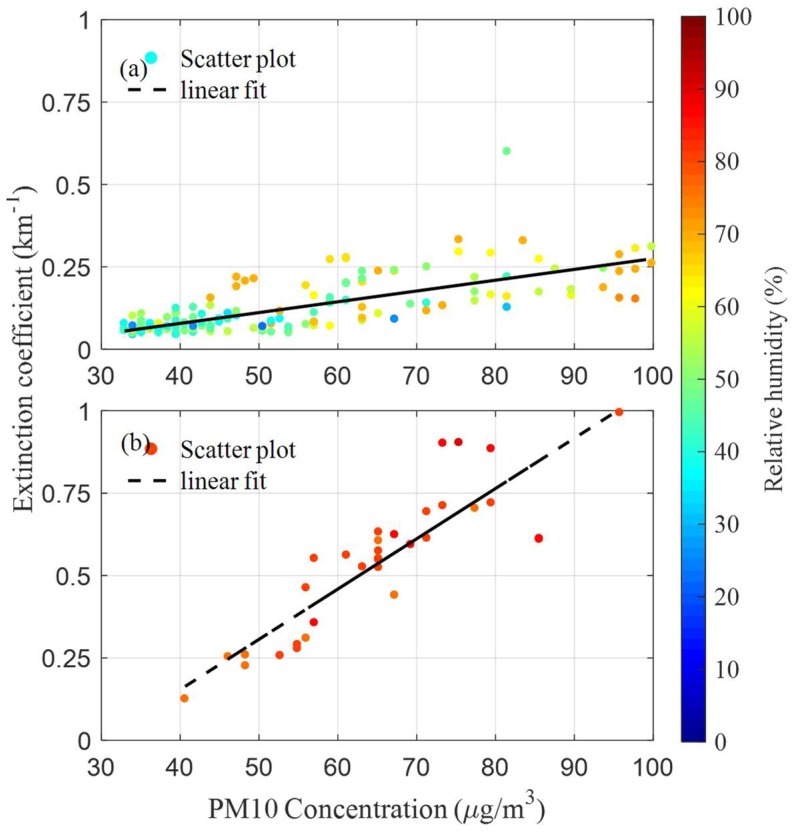
The correlation between the aerosol extinction coefficient and the PM10 concentration when the PM10 concentration is lower than 100 µg/m^3^, the relative humidities are: (**a**) ≤75% and (**b**) >75%, respectively.

The spatial-averaged aerosol extinction coefficient was further averaged in one hour, as the particle concentrations were reported once-an-hour by the national monitoring station. The relationship between the one-hour averaged aerosol extinction coefficient and the PM10 concentration with different relative humidities is shown in Figure 7. Generally speaking, the aerosol extinction coefficient increased with the increasing of the particulate concentrations. However, the aerosol extinction coefficient was also influenced by atmospheric relative humidity and particle compositions, etc. As shown in Figure 8, the coefficients of the linear fitting between the aerosol extinction coefficient and the PM10 concentration are quite different under different relative humidities when the PM10 concentration is below 100 µg/m^3^. The coefficient of the linear fitting is 0.015 km^−1^/µgm^−3^ in the case of high relative humidity (>75%), while it is only about 0.0033 km^−1^/µgm^-3^ when the relative humidity is no more than 75%. The correlation coefficients between the aerosol extinction coefficient and the PM10 concentration in the case of high relative humidity (>75%) and low relative humidity (≤75%) are 0.88 and 0.73, respectively. On the other hand, the discrepancy of the aerosol extinction coefficient under high and low humidities is not significant when particle concentrations are beyond 120 µg/m^3^. The relationship between the aerosol extinction coefficient and particulate concentration is more sophisticated.

## 5. Conclusions

This work developed a 450-nm Scheimpflug lidar system for atmospheric pollution monitoring by employing a 3.5 W 450-nm continuous-wave laser diode and a CMOS image sensor. 24-h continuous atmospheric monitoring on a near horizontal path in December 2017 is achieved with a 10-nm FWHM interference filter to suppress the sunlight background. The laser beam of the laser diode, which has large divergences along the fast and the slow axes, is collimated by a cylindrical lens pair and a F6 achromatic refractor, to improve the geometrical efficiency. The laser power that is transmitted into atmosphere is estimated to be 2.7 W. The divergence of the laser beam in the Scheimpflug plane is about 0.1 mrad. The SNRs of the lidar signals were beyond 150 and 300–400 for daytime and nighttime, respectively. The noise of the lidar signal in daytime measurements is dominated by the sunlight background and may be further suppressed by employing a narrowband interference filter. However, it is limited by the PRNU noise of the image sensor during nighttime measurements. The SNR can be further improved by employing image sensors with lower PRNU noise, e.g., scientific CMOS or CCD sensors. The PRNU noise could be as low as 0.01%. The promising result also implies that differential absorption lidar (DIAL) monitoring of atmospheric nitrogen dioxide (NO_2_) can be feasible during daytime based on the present 450-nm lidar system with improved SNRs.

The aerosol extinction coefficient is extracted from lidar data by the Fernald inversion method, which is then spatially averaged to compare with local particle concentrations. It has been found that atmospheric relative humidity has played a significant role on the aerosol extinction coefficient due to hygroscopic growth of particles. Experimental result reveals that the linear ratio between the aerosol extinction coefficient and PM10 concentration under high relative humidity (75–90%) is about two-times that in low relative humidity (≤75%) when PM10 concentrations are less than 100 µg/m^3^. Nevertheless, the relationship is more sophisticated with the increasing of the relative humidity and particle concentrations.

## Figures and Tables

**Table 1:** Primary specifications of the 450-nm Scheimpflug lidar (SLidar) system.

	Model	Specifications
Laser source	Nichia, NDB7K75	Wavelength: 450 nm; Power: 3.5 W; Divergence: 14° ║ × 46° ┴;
Collimator	Tianlang, F6 refractor	Focal length: 600 mm, Diameter: 100 mm
	Cylindrical lens pair: LJ1918L1-A & LK1426L1-A	Convex lens: f = 5.8 mm, height: 4 mm; Concave lens: f = −25 mm, height: 10 mm Laser beam divergence: 0.1 × 0.36 mrad
Receiver	Skywatcher, CFP200	Focal length: 800 mm; Diameter: 200 mm
Detector	CMOS, CMV2000 Lt225NIR	Tilt angle: 45°; Resolution: 2048 × 1024 Pixels; Frame rate:170 fps; Bit depth: 12/8 bit; Pixel size: 5.5 µm × 5.5 µm; Quantum efficiency: 45% @ 450 nm; ROI: 2048 × 200 pixels
Filters	450 nm interference filters	10 nm FWHM (Edmund optics)
